# Christmas Festival at the Hospitals

**Published:** 1887-01-08

**Authors:** 


### CHRISTMAS FESTIVAL AT THE HOSPITALS.

By our Special Commissioner.

### THE PADDINGTON GREEN CHILDREN'S HOSPITAL.

Father Christmas in his Element.

In few places does Father Christmas put in a cheerier appearance than in the London hospitals, and, of all the London hospitals, those devoted to children are the ones in which one fancies he comes with a kindlier face and a more abundant generosity than to any others. So, at least, the visitor is inclined to think as he steps into one of the wards of Paddington Green Hospital for Children, and looks round upon the preparations for the Christmas entertainment. Among the tiny patients here there are cases of terrible affliction—many of them such as render a fervent wish that Death may speedily do his work, the truest charity—but Father Christmas has done his best to make even the most afflicted of them forget for a while that it is for them a world of sadness and suffering, to persuade them that there is, after all, much that is bright and beautiful. Indeed, one's first impression of this small hospital in its festive array is not that it is a retreat for the suffering, but a cheerful, pleasant home, gay with everything that can fill childish eyes with wonder, and make childish hearts dance for joy.

Its true Significance.

It is only when one goes from bed to bed, and inquires a little into the cases of the little occupants, that the true significance of even the smallest of children's hospitals begins to smite upon the heart, and to make the very sunbeams that are stealing in upon them heavy with sorrow. There, under the window, lies a little thing six years old—a rosy-cheeked, brilliant-eyed, gentle-looking child, clad, as they all are, in a little scarlet tunic. One side of the face is somewhat swollen, else one might suppose that she was hearty and well, and would by-and-bye be among the liveliest of the dancers around the gorgeous Christmas-tree there. Alas! she will never dance. She lies there on her back with a bad spinal disease; she has an abscess on her arm; she has lost one finger, and has had half of her jaw removed.

Some Patients.

Over in the other corner yonder is a noisy little rogue, whose exuberance of spirits has occasionally to be checked as he rolls about in his bed and gazes up at the glittering tree that almost overshadows him. He, too, has lost a finger; has disease of the knee and ankle, and has a head of an abnormal size, caused probably, by abscesses. On the other side the Christmas-tree is a little thing turning and tossing, with strange spasmodic movements, due to St. Vitus's dance; and a little farther along is an unfortunate little creature terribly disfigured with eczema. Still more doleful to look at is a sad-faced little fellow, who "always cries when a stranger approaches him," and whose normal expression of face is that of a child who finds he has made a lamentable mistake in coming into this world at all, and would be glad to withdraw if he only knew how. Even a cheery allusion to the world of toys around him, the pictures, the flowers and the evergreens, and the coming festivity, hardly avails to keep back the tears of this pessimistic patient of five. Next comes one too ill to heed any of the gaiety around, a little girl of six, with hopeless heart-disease. With purple face and weary eye, she lies and pants and waits for release. God grant that it may be soon! In the next cot, sweetly sleeping behind a pictured screen, is a tiny fair-haired child of two, a beautiful baby, who has fallen off a stool and broken a leg; and near at hand is a patient who, strictly speaking, ought not to be here, but, poor little mannikin, he has lain here till he cannot be moved. One fancies that he, too, is eagerly scanning the festive preparations, and speculating on which of those wonderful toys, or how many of those glittering bonbons will fall to his share. But, no; he is "blind Tom." Those brilliant eyes of his are rolling in darkness, and darkness, too, is gradually stealing over brain and sense. He has lain here for two years, blind and paralysed, and he used to be immensely popular with those familiar with the institution; but tumours are forming on the brain, and the childish playfulness is fading out, and poor little Tom is growing dull and uninteresting. By-and-bye, lights will flash from the Christmas-tree, but never a ray will reach poor Tom; and the magic-lantern will display its wonders all in vain for him; while even Punch and Judy will fail utterly to evoke any enthusiasm on his part.

Convalescent Children.

Mingled with them and other sad and hopeless little patients are many whose ailments are not so serious, and for whom all the preparations that have been going on for days have had inexpressible interest. The wards are literally ablaze with toys and flags and artificial flowers; the walls are beautifully festooned with evergreens, and in every way all that profuse liberality and ungrudging personal devotion on the part of officers and friends of the institution can do to bring home to these tiny sufferers something of the happiness that at this time of year gladdens so many who are in health and strength outside, has been done. The last touches are being given, and in two or three hours festivities will commence.

### UNIVERSITY COLLEGE HOSPITAL.

A Contrast.

Not prettier, not more indicative of earnestness and hearty devotion, but far more extensive and imposing were the preparations proceeding at the same time for a grand entertainment at the University College Hospital on the same evening—Wednesday, the 29th December, that is to say. The large institution in Gower Street was metamorphosed almost out of recognition, and the decorations of one sort or another, and the Christmas gifts, must not only have cost considerable sums of money, but must have involved immense labour. Here, however, as at Paddington Green, everything in the shape of festivity was provided out of funds specially contributed for the purpose, or by the voluntary labours of the hospital staff and friends interested in the establishment. A really very fine decorative display was thus got up without the cost of a penny to the hospital funds. The corridors on the ground-floor were very prettily adorned by Messrs. Maple and Co., who, in response to an appeal to them, not only gave what they were asked for—the materials—but sent workmen to put them up. Everywhere wards were festooned with evergreens, walls were decorated with tasty and elaborate tablets, mottoes, groups of flags, and so forth; and on all hands were toys in such abundance, that one might have supposed that all the patients in the place had renewed their youth. The main show of these, however, was in the children's ward on the upper floor, which really looked on Wednesday quite charmingly attractive and pleasant; and though, if one were to go round from bed to bed, he would find here of course, cases just as pitiably sad as those already alluded to in connection with Paddington Green, there were so many in this ward getting on toward recovery, or sufficiently well to be thoroughly interested in what was going forward, that it was the festivity and brilliancy of the occasion that one saw, and what was painful was all but hidden. In about a dozen cases children who had recently been discharged, quite recovered, had been specially invited to the entertainment. The poor little folks had seen and heard so much about the grand festival at Christmas that it seemed hard to turn them out just as the excitement of the thing was reaching a climax, and the grand event about to come off. So they were invited, and very much at home they evidently found themselves, and highly enjoying the unwonted splendours that had unfolded all around them. The grand acme of all that was beautiful in this ward was a really exquisite doll, as large as some of the patients, and beautifully dressed. This had been sent by *Truth*, and was decidedly too good for a workaday world like this. In fact, it was reserved to be shown as a sort of Queen of the Feast at times of rejoicing.

Several Christmas Trees.

Of Christmas-trees there were several; and presents of almost every imaginable description, from umbrellas and reading-lamps, to pairs of trousers and railway rugs, were displayed in various parts of the building. These, like all the rest of the day's good things, were provided either by gift of the things themselves, or out of funds raised for this especial purpose. It is the kindly custom of the place to make all the inmates, and all the humbler officers of the establishment, presents at Christmas time, even down to the "scrubbers," for whom pieces of beef are provided. For the patients, articles are, as far as possible, selected to meet their requirements. A man who needs a pair of trousers will have a pair given him, or if boots be the needful thing, it will be boots. Everybody would get something, and the array of presents on Wednesday made a brave show. The most striking objects in the wards, however, were a dozen table decorations—really cleverly fabricated little representations of some scene of fiction or of real life. On one table was an Indian Temple, a most elaborate piece of work, executed in pith, and presenting in its vicinity a pleasingly realistic representation of palm-trees and sand, native Hindoos with their merchandise, their elephants, and so forth. In another ward was a Cumberland valley, with its thickets of trees, and a church, beside a running stream. Another was Robinson Crusoe's Island, with Crusoe in his hut, and the savages on the coast. A scene from Shakespeare's *Tempest* was the subject of another, and the rocks and the sea-beach, and the enchanted cave, cunningly contrived out of hearthstone, and with bush and shrubs, water of looking-glass, and real shingle and seaweed from Eastbourne, were all wonderfully well done. Another scene represented a party of snowballers; and on another table was an extensive and highly appreciated presentment of Jack and the Beanstalk. All these things, largely made up of flowers and shrubs and thickets of moss, were wonderfully effective as decorations, and, it appeared, were the work of the "sisters" and nurses and students.

Generous Corney Grain.

The entertainment began at five o'clock, and a large number of invited guests, and such of the patients as were equal to it, were very pleasantly entertained for some hours. Mr. Corney Grain very kindly gave, gratuitously, "A Musical Sketch" in one ward, while in another Mr. Horace Chester amused an audience of patients and guests by "A Musical and Mimetic entertainment." At seven o'clock there was a grand concert. The children were separately provided for in their own ward, where a Punch and Judy performance afforded a deal of fun. Altogether, it appears to have been a very successful evening, so far as enjoyment was concerned. It is to be hoped that the festival will prove to have been of substantial service to the funds of the hospital, which, like most of the others, is in dire straits.

